# Persistent Symptoms After SARS-CoV-2 Infection in a Referred Occupational Clinical Registry: Symptom Patterns and Associated Factors

**DOI:** 10.3390/diseases14040141

**Published:** 2026-04-09

**Authors:** Agnessa Kozak, Jana Wischnat, Corinna Rademacher, Andreas Gonschorek, Ingo Schmehl, Susann Seddigh, Andrea Fürst, Kai Wohlfarth, Lynn Engel, Jakob Wefers, Kerrin Kobes, Olaf Kleinmüller, Majid Essa, Martin Tegenthoff, Albert Nienhaus, Peter Schwenkreis

**Affiliations:** 1 German Social Accident Insurance Institution for the Health and Welfare Services (BGW), D-22089 Hamburg, Germany; jana.wischnat@bgw-online.de (J.W.); albert.nienhaus@bgw-online.de (A.N.); 2 Department of Neurology, BG Hospital Bergmannsheil, D-44789 Bochum, Germany; corinna.rademacher@bergmannsheil.de (C.R.); lynn.engel@bergmannsheil.de (L.E.); jakob.wefers@bergmannsheil.de (J.W.); martin.tegenthoff@rub.de (M.T.); peter.schwenkreis@rub.de (P.S.); 3 Department of Neurology, BG Hospital Hamburg, D-21033 Hamburg, Germany; a.gonschorek@bgk-hamburg.de; 4 Department of Neurology, BG Hospital Berlin, D-12683 Berlin, Germany; ingo.schmehl@ukb.de; 5 Department of Neurology, BG Hospital Duisburg, D-47249 Duisburg, Germany; susann.seddigh@bg-klinikum-duisburg.de; 6 Department of Neurology, BG Hospital Murnau, D-82418 Murnau, Germany; andrea.fuerst@bgu-murnau.de; 7 Department of Neurology, BG Hospital Bergmannstrost, D-06112 Halle, Germany; kai.wohlfarth@bergmannstrost.de (K.W.); majid.essa@bergmannstrost.de (M.E.); 8 Medical Faculty, Health and Medical University Erfurt (HMU), D-99084 Erfurt, Germany; 9 Centre for Epidemiology and Health Services Research for Healthcare Professionals (CVcare), University Medical Centre Hamburg-Eppendorf (UKE), D-20246 Hamburg, Germany; k.kobes@uke.de (K.K.);; 10 Center for Theoretical and Integrative Neuro- and Cognitive Science (THINK), Ruhr-University Bochum, D-44803 Bochum, Germany

**Keywords:** Post-COVID-Syndrome, persistent symptoms, occupational diseases, multicenter study, health outcomes, healthcare and welfare personnel

## Abstract

Background/Objectives: Persistent symptoms following SARS-CoV-2 infection pose a substantial burden in occupational settings. This study aimed to characterize symptoms following work-related SARS-CoV-2 infection and to assess their associations with sociodemographic and clinical factors. Methods: Data were obtained from a multicenter clinical registry of insured individuals referred for persistent symptoms 12 weeks after laboratory-confirmed work-related SARS-CoV-2 infection. Participants were assessed within a standardized post-COVID diagnostic program at six specialized clinics for occupational accident insurance in Germany. Persistent symptoms reported by ≥50% of participants were analyzed using generalized linear mixed models with random intercepts for center. Results: A total of 1511 participants (76.7% women; median age 54 years) were included, with a median interval of 16 months between infection and assessment. On average, participants reported ten persistent symptoms. The most frequent complaints were limited physical capacity (95.6%), concentration difficulties (78.8%), dyspnea (70.5%), exhaustion/tiredness (68.9%), and memory difficulties (67.5%). Individuals reporting more than ten acute symptoms had increased odds of persistent complaints (ORs between 2.1 and 4.66). Hospitalization was independently associated with persistent dyspnea (OR 1.62; 95%CI 1.17–2.25). Reinfections were linked to exhaustion and cognitive fatigue. Compared with Omicron, wild-type infection was associated with higher odds of concentration difficulties (OR 1.65; 95%CI 1.17–2.33). Comorbidities demonstrated symptom-specific associations. Conclusions: Among individuals with work-related SARS-CoV-2 infection, limited physical capacity and cognitive impairments were the most frequently reported symptoms, and higher acute symptom burden was strongly associated with the development of persistent symptoms. These findings support course-oriented evaluation and symptom-specific approaches in occupational disease assessment and management.

## 1. Introduction

From the outset of the pandemic, it became evident that certain occupational groups were at increased risk of SARS-CoV-2 infection and disease [1,2]. In particular, workers in system-critical, person-centered occupations, such as healthcare and nursing, social services, education, and protective services, were disproportionately affected [3,4]. In Germany, COVID-19 is recognized as an occupational disease (No. 3101) if the insured persons were engaged in professional activities in healthcare, welfare services, laboratory work, or other occupations with a comparably high risk of infection. COVID-19 cases are also classified as occupational accidents if the insured persons were also engaged in a covered occupational activity and a clearly documented close contact with an index case occurred within a defined time period [5]. By 30 June 2025, the institutions of the German Social Accident Insurance (DGUV) had recognized 370,215 occupational disease cases and 27,195 occupational accidents attributable to COVID-19. In total, 351 deaths related to COVID-19 were documented as occupational diseases or occupational accidents [6]. While most individuals recover within a few weeks, in some cases, symptoms persist for more than three months or new symptoms occur that cannot be explained by alternative diagnoses. This is referred to as post-COVID syndrome (PCS) [7].

Due to the substantial heterogeneity of PCS, its prevalence is difficult to determine and depends on the definition applied, the data collection methods used, the follow-up duration, and the composition of the study population [8]. Healthcare workers are particularly affected, with recent meta-analyses reporting prevalence rates of approximately 40–42% for persistent symptoms [9,10,11,12]. However, the presence of a control group is crucial. Since the reported symptoms are nonspecific, studies without a control group may overestimate the prevalence [13]. Commonly reported symptoms include fatigue, exercise intolerance, cognitive impairment, shortness of breath, sleep disturbances, and musculoskeletal pain [14,15]. Individuals affected by these symptoms report higher levels of psychological distress (including depression and anxiety) and a reduced quality of life, particularly with respect to physical domains [16,17]. Moreover, Rahmati et al. found that approximately one in seven individuals remained unable to work even two years after infection [10].

A severe acute course of COVID-19 requiring hospitalization, as well as risk factors such as older age, female sex, pre-existing comorbidities, and pronounced inflammatory responses, have been shown to increase the risk of persistent physical and psychological impairments [10,18,19]. A longitudinal analysis of insured individuals with COVID-19 reported as an occupational disease demonstrated that older age, female sex, multimorbidity, respiratory and hormonal/metabolic comorbidities, as well as a higher number of acute symptoms, were significant risk factors for symptom persistence over time [20]. In addition, the study by Kahlert et al. identified infection with pre-Omicron variants as a strong predictor of PCS; affected individuals reported significantly higher levels of fatigue and depressive symptoms and rated their general health status substantially worse [21]. In line with this, Ballouz et al. showed that infection with the Omicron variant among previously vaccinated individuals was associated with a significantly lower risk of PCS compared with infection with the wild-type virus in unvaccinated individuals [22]. Severe COVID-19 courses, particularly those involving hospitalization, are considered key predictors of PCS, regardless of the viral variant [23,24]. Although overall morbidity and mortality risks have declined over time, mortality risk among hospitalized patients remains elevated even three years after infection (RR 1.29; 95% CI: 1.19–1.40) [23]. However, persistent symptoms may also occur in individuals who did not require hospitalization [24]. Psychological factors may also play an important role in the persistence of physical symptoms after a case of mild acute COVID-19 [19,25]. The duration of recovery is closely related to the severity of the acute infection, with more severe courses being associated with longer-lasting symptoms. While symptoms resolve within one year for the majority of individuals, a substantial proportion continue to experience ongoing complaints [14]. For this group, the long-term disease trajectory remains insufficiently understood.

Given the partially prolonged and highly individual trajectories observed, identifying factors associated with the persistence of specific symptoms is of particular relevance. To improve understanding of the manifestation and distribution of persistent symptoms, the present study focuses on subjectively reported complaints at the time of outpatient or inpatient assessment within the post-COVID diagnostic program.

This study aimed to comprehensively characterize subjectively reported persistent symptoms after work-related SARS-CoV-2 infection and to assess their relationships with sociodemographic and clinical characteristics. Emphasis was given to potential differences associated with sex.

## 2. Materials and Methods

### 2.1. Study Design and Setting

This is a multicenter study that uses a prospective referred clinical registry, complemented by retrospectively recorded information. The specialized BG hospitals of the German Statutory Accident Insurance established an interdisciplinary diagnostic program for insured individuals with persistent, work-related post-COVID symptoms. Patients were managed according to symptom severity: mild cases were evaluated on an outpatient basis, whereas more complex cases with potential risk of prolonged work incapacity were admitted for an inpatient “Post-COVID Check” (PCC), involving extensive cross-disciplinary diagnostics. The clinical findings informed individualized therapeutic and rehabilitation strategies, accompanied by follow-up assessments. All diagnostic data were incorporated into a centralized patient registry across six participating BG hospitals. Comprehensive methodological details regarding the study design and data acquisition were published in a primary paper by Schwenkreis et al. [26].

### 2.2. Participants and Eligibility Criteria

Eligibility for the post-COVID registry was restricted to individuals aged 18 years or older who exhibited persistent or newly emerging symptoms persisting for more than 12 weeks after a laboratory-confirmed SARS-CoV-2 infection acquired in the workplace. The preceding infections had to be officially recognized either as an occupational infectious disease or as an occupational accident [5]. Participants were assessed in either outpatient or inpatient settings at one of the six BG hospitals. Eligible individuals represent diverse professional backgrounds and have provided informed consent for the use of their clinical data. No explicit exclusion criteria were specified.

Recruitment for the post-COVID registry was conducted both prospectively and retrospectively. As part of the DGUV rehabilitation management process, insured individuals were referred for diagnostic evaluation to one of the participating BG hospitals upon recommendation by the accident insurance providers. After receiving detailed information and providing informed consent, they were prospectively enrolled in the registry, which commenced on 17 August 2021. Individuals who were evaluated before the registry was initiated were contacted retrospectively to obtain consent for the use of their data. Follow-up assessments are planned and will be carried out as needed, particularly after rehabilitation interventions. A quality-controlled data extract comprising all cases entered into the registry up to 30 December 2024 was used for the present analyses. Procedures and results are reported in accordance with the STROBE guidelines for cohort studies [27].

### 2.3. Data Collection

Given the heterogeneity of PCS, routine clinical data are recorded in a symptom-driven manner. The diagnostic process starts with a standardized medical history covering demographic characteristics, occupational and social background, and the course of SARS-CoV-2 infection, followed by further clinical, instrumental, and imaging investigations based on individual needs. During the clinical interview, patients were asked about symptoms experienced during acute infection as well as persistent symptoms at the time of clinical assessment. Symptom assessment was based on self-reported outcomes obtained during the clinical interview. Physicians at all centers were provided with a standardized, pre-specified case report form (CRF) listing 32 predefined symptom categories for use as a structured guide during the clinical interview [26]. The CRF ensured consistent symptom elicitation across centers. Symptoms were recorded as binary variables (present = “yes”/absent = “no”). Further complaints not captured by predefined categories were additionally recorded as free-text entries, which were subsequently checked and, where appropriate, mapped to existing symptom categories. Limited physical capacity was operationalized as patient-reported reduction in physical performance or early exhaustion during daily and work activities compared with the pre-infection state. Furthermore, impaired concentration was defined as difficulty maintaining attention during everyday cognitive tasks, whereas cognitive fatigue referred to concentration problems that occur and worsen after sustained mental effort and require a longer recovery period. No additional standardized performance or neuropsychological testing was used to operationalize these outcomes for the present analyses.

All clinical information was initially documented in the local clinical information system (MEDICO) and physician reports. For research purposes, the data were subsequently transferred into electronic CRFs within the Research Electronic Data Capture (REDCap) developed by the Vanderbilt University REDCap platform [28]. To ensure consistent data recording across centers, study nurses were handed a data entry manual and underwent training in its use at study initiation. Regular meetings among site coordinators were held throughout the data collection period to maintain procedural consistency. Standardized and automated data quality procedures were applied to ensure completeness and plausibility of registry data across centers.

Further information was collected on sociodemographic characteristics such as age and sex, weight, height, occupation and workplace, the course of the acute illness (date of initial infection and reinfections, SARS-CoV-2 detection technique, hospitalization, intensive care treatment (ICU) and length of stay, symptoms), pre-existing conditions and vaccination status (vaccination dates, number of doses, and manufacturer). However, vaccination status could not be obtained for all participants, particularly for those enrolled during the early phases of the registry, when vaccination campaigns had only recently begun and documentation was not yet consistently implemented across centers.

### 2.4. Statistical Methods

Descriptive statistics were used to characterize the study sample. Group comparisons were performed using chi-square or Fisher’s exact tests for categorical variables and the Mann–Whitney U test for continuous variables.

As participants were exclusively individuals referred for post-COVID occupational health assessment due to persistent complaints, the reported frequencies reflect the symptom burden of this clinically selected population. Persistent symptoms reported by at least 50% of participants were defined as dependent variables for the present analyses. Although headache met the predefined frequency criterion (52%), it was not included in the present analysis, as it was examined in a separate focused evaluation. The independent variables examined included age, sex, occupation, BMI category, hospitalization due to acute COVID-19, time between infection and clinical assessment, infection wave (based on date of primary infection), number of symptoms during the acute phase, number of reinfections, number of pre-existing conditions, as well as specific comorbidity groups (cardiovascular, neurological, psychiatric, pulmonary, metabolic and other disorders) and prior utilization of rehabilitation services. Vaccination status was not included in the models due to substantial missing data resulting from inconsistent documentation across centers (42% missing). To assess the robustness of the main findings, sensitivity analyses were conducted among participants with available vaccination data. Within this subset, models were estimated with and without adjustment for vaccination status. Adjustment for vaccination status did not change the estimated effects of the independent variables meaningfully (Supplementary Tables S1–S8). Data entries with the response option “no information” were recoded as missing values and handled accordingly in the analysis. The proportion of missing data for all independent variables is provided in Supplementary Table S9. No missing data occurred in the dependent variables.

For each outcome, theoretically relevant variables associated at *p* < 0.10 in bivariate analyses were entered into outcome-specific initial generalized linear mixed models, including a random intercept for center. An overview of the bivariate analyses is provided in Supplementary Table S10. Model reduction was conducted using backward elimination based on likelihood ratio tests and changes in Akaike information criterion (AIC) and Bayesian information criterion (BIC). For each full model, a complete-case dataset was constructed and used for all nested model comparisons to ensure identical observations across models. Variables were retained if removal worsened model fit or conflicted with theoretical plausibility. Sex and age were included as covariates due to theoretical relevance and their role as potential confounders, regardless of statistical significance. Interaction terms were explored but not incorporated in the final models due to a lack of statistical significance.

For the outcome limitation of physical capacity, a Bayesian logistic regression model was fitted due to substantial outcome imbalance (95.6% positive cases). This approach allows the inclusion of priors, which can help stabilize parameter estimates and reduce the risk of inflated odds ratios in sparse settings [29,30]. Weakly informative priors were specified for fixed effects (normal (0, 1.5)), the intercept (normal (logit (0.96), 1.5)), and the random-intercept standard deviation (exponential (2)). This provides regularization comparable to penalized frequentist approaches while maintaining hierarchical modeling. The model was estimated using the brms package (version 2.23.0) in R with four Markov chains (4000 iterations each, 1500 warm-up). Convergence diagnostics indicated stable posterior sampling (Rhat ≈ 1.00 with large effective sample sizes).

Results are reported as odds ratios (ORs) with 95% confidence intervals (CI; frequentist model) or 95% credible intervals (CrI; Bayesian model) [31].

Descriptive analyses were performed using SPSS (version 28; IBM, Armonk, NY, USA), and the regression analyses and figures were conducted using R (version 4.3.2) [32]. Generative AI (OpenAI, model GPT-5.2) was used to translate segments into English and to shorten texts.

## 3. Results

### 3.1. Demographic and Clinical Data

**Figure 1:**
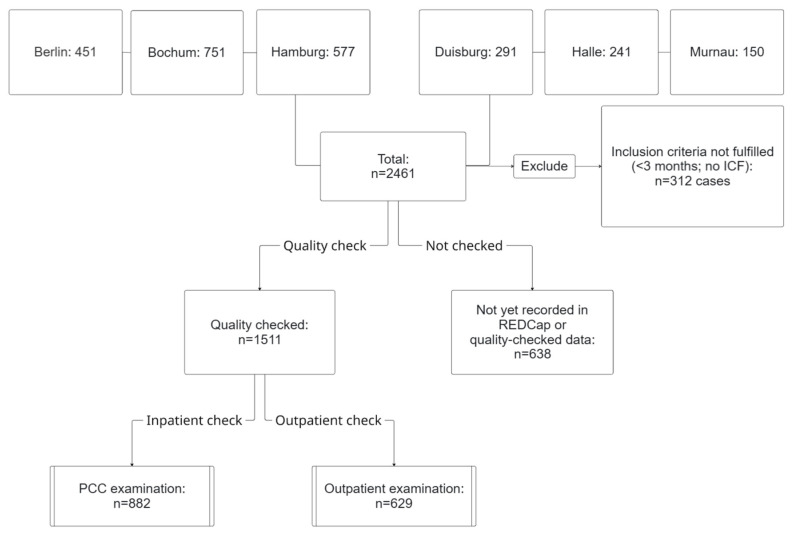
Flowchart of participant inclusion across six centers. Of 2461 documented cases, 1511 participants with quality-checked data met the inclusion criteria and were included in the analyses (882 inpatient, 629 outpatient). Abbreviation: ICF, Informed Consent Form.

Of the 2149 eligible patients, 2028 from the six participating hospitals met the inclusion criteria and were enrolled in the registry study. As patient recruitment and examination are ongoing, not all case data has been entered or quality-checked in the REDCap electronic database (Figure 1). Therefore, data from 1511 patients were available for analysis, of whom 76.7% were female. The median age at examination was 54 years (IQR 44–59). The median time between infection and clinical examination was 16 months (IQR 11–22). At the time of examination, about half of the participants (51.8%) were unable to work. Based on body mass index (BMI), 66.7% were overweight or obese. The majority worked in hospitals (34.6%) or in educational and social welfare institutions (22.9%). Most women worked in nursing (48.7%) or educational and social professions (24.7%), whereas men were predominantly employed in administrative or technical positions (47.1%). Pre-existing conditions were common in both sexes, with 63% having two or more comorbidities. Cardiovascular diseases were significantly more common among men (47.2 vs. 37.3%), whereas psychiatric (15.9 vs. 23.4%), neurological diseases (14.8 vs. 20.9%), allergies (29.5 vs. 41.2%) and autoimmune disorders (2.8 vs. 6.0%) were significantly more frequent among women. Vaccination status was available for 873 participants, 87% of whom had received at least one COVID-19 vaccination. Based on available vaccination and infection dates, vaccination status prior to the first infection could be determined for part of the cohort: 53% were unvaccinated, 12% vaccinated, and data for the remaining 35% were missing.

**Figure 2:**
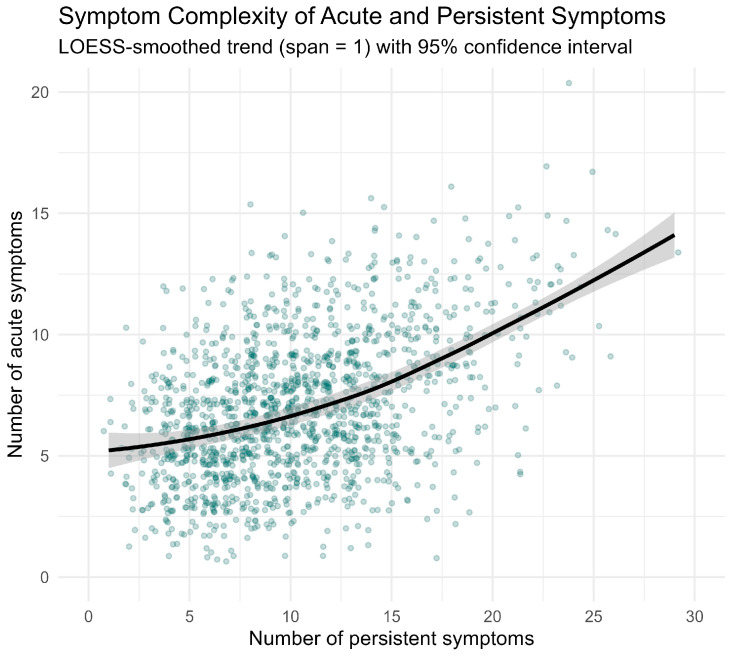
Relationship between the number of symptoms reported during the acute infection phase (*y*-axis) and the number of persistent post-COVID symptoms (*x*-axis) at clinical assessment. Each dot represents one participant. The solid line shows a LOESS smoothed trend (span = 1) and the grey shaded area indicates the 95% CI.

Based on the time of initial infection, most infections occurred during the period when the wild-type variant was dominant (63.1%), followed by Omicron (17.9%), Alpha (12.8%) and Delta (6.2%). A reinfection was documented in 13.4% of participants, most of them with the Omicron variant. During the acute phase of infection, 19.6% of participants required hospitalization, and 5.1% were treated in the ICU. Compared to women, men were hospitalized significantly more often (31% vs. 16%) and more frequently required intensive care (12% vs. 3%), indicating a more severe disease course among male participants (Table 1). Most participants experienced a moderate number of acute symptoms at first infection, with 61.2% reporting 5–9 symptoms and 18.6% ten or more. The most frequently reported symptoms were flu-like, including fever and chills (69.3%), cough (67.6%), limb pain (65.8%), shortness of breath (64.2%), headache (64%), exhaustion/tiredness (61.7%) and loss of taste and smell (52.6%). Severe organ involvement was reported in 9.8% of cases, including pneumonia, pulmonary embolism, renal failure, myocarditis, severe acute respiratory distress syndrome (ARDS) and bacterial superinfection. Peripheral nervous system involvement (e.g., facial palsy or polyneuropathies) was reported in 1.1% of cases, and in 0.3%, the central nervous system (e.g., encephalitis, myelitis) was affected. A higher acute symptom burden was associated with a greater number of persistent complaints (Figure 2). Individuals reporting five to nine acute symptoms had an average of 9.9 (SD 4.1) persistent symptoms, whereas those with ten or more acute symptoms reported an average of 13.9 (SD 5.1) symptoms.

### 3.2. Frequency of Reported Persistent Symptoms

**Figure 3:**
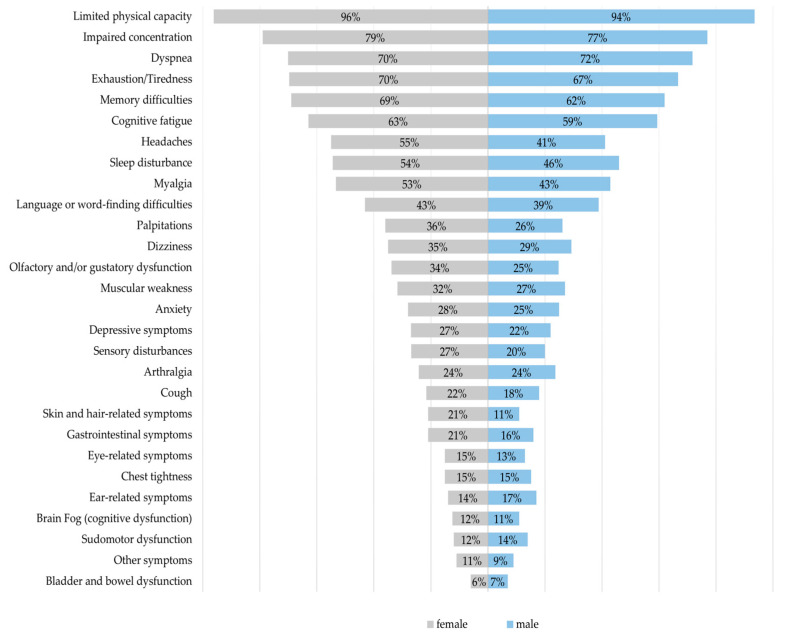
Sex-stratified proportions of participants reporting persistent symptoms at clinical assessment. The percentages indicate the frequencies of participants reporting each symptom (multiple responses). Symptoms are ordered by decreasing frequency among females.

A total of 55 different persistent symptoms were documented during the clinical interviews. On average, patients reported ten (SD 4.6) subjectively perceived complaints. The most frequently reported symptoms were limited physical capacity (95.6%), impaired concentration (78.8%), dyspnea (70.5%), exhaustion/tiredness (68.9%), and memory difficulties (67.5%). More than half of the participants additionally reported cognitive fatigue (62.2%), sleep disturbances (52.6%), headaches (51.7%), and myalgia (50.8%). Psychological symptoms such as anxiety or depressive symptoms were present in approximately one in four patients. The average symptom burden was significantly higher among women (M = 10.6; SD 4.5) compared with men (M = 9.4; SD 4.7). Women also exhibited significantly higher proportions of reported headaches, sleep disturbances, myalgia, palpitations, olfactory and gustatory dysfunction, and hair loss or dermatological complaints (Figure 3).

### 3.3. Regression Analyses

Table 2 shows the results of a multivariable analysis of the most commonly reported post-COVID symptoms during the examinations. Across all outcomes, acute symptom burden at first infection was the most consistent predictor. Those who reported more than 10 symptoms had significantly higher odds for all outcomes, varying between OR 2.1 and OR 4.66. Having even 5–9 acute symptoms was associated with higher odds for concentration difficulties (OR 1.54; 95% CI 1.12–2.10), dyspnea (OR 1.69; 95% CI 1.26–2.25), exhaustion (OR 1.46; 95% CI 1.10–1.93) and cognitive fatigue (OR 1.47; 95% CI 1.12–1.93). A severe acute disease course, as indicated by hospitalization, was found to be independently associated with persistent dyspnea (OR 1.62; 95% CI 1.17–2.25). A history of reinfection was associated with an increased risk of experiencing exhaustion/tiredness (OR 1.54; 95% CI 1.07–2.22) and cognitive fatigue (OR 1.69; 95% CI 1.20–2.39). Compared to patients infected with the Omicron variant, those infected with the wild-type SARS-CoV-2 variant were significantly more likely to experience concentration difficulties (OR 1.65; 95% CI 1.17–2.33).

After controlling for other variables, sex was not consistently associated with all outcomes. However, women were found to be more likely to experience myalgia (OR 1.47; 95% CI 1.14–1.89). There was only a limited association between age and persistent symptoms, although patients aged 50–59 years were more likely to experience sleep disturbances (OR 1.45; 95% CI 1.05–1.99). Higher BMI was associated with dyspnea (OR 1.46; 95% 1.08–1.97) and memory difficulties (OR 1.42; 95% CI 1.07–1.87).

Moreover, comorbidities showed symptom-specific associations. Pulmonary conditions were linked to dyspnea (OR 1.70; 95% CI 1.26–2.29), while psychiatric conditions were associated with both concentration difficulties (OR 1.50; 95% CI 1.02–2.19) and exhaustion/tiredness (OR 1.58; 95% CI 1.17–2.13). Cardiovascular conditions were related to myalgia (OR 1.35; 95% CI 1.08–1.70), whereas neurological and metabolic conditions were associated with sleep disturbances (OR 1.32; 95% CI 1.00–1.74 and OR 1.48; 95% CI 1.17–1.88, respectively). Furthermore, an increasing number of pre-existing conditions was associated with a higher likelihood of concentration difficulties, among both individuals with two to three conditions (OR 1.61; 95% CI 1.20–2.16) and those with four or more conditions (OR 2.35; 95% CI 1.57–3.53).

## 4. Discussion

Data from this registry study within the BG clinical network revealed substantial complexity of persistent symptoms following work-related SARS-CoV-2 infection, predominantly among health and social care workers. The average duration of symptoms was 16 months after confirmed SARS-CoV-2 infection, underscoring the long-term burden of post-COVID conditions in these occupational groups. While men were more likely to experience a severe acute course in this cohort, women reported a greater overall number of persistent symptoms. The findings indicate that a high degree of symptom complexity during the acute phase is closely associated with a more complex and persistent disease course. The results further demonstrate that pre-existing conditions, hospitalization, viral variant, reinfections and body mass index were key factors associated with persistent physical and cognitive complaints. Sex-specific differences and age-related effects were observed selectively.

Patients who present for a post-COVID check-up or outpatient consultation often report a variety of persistent or newly occurring symptoms. Nearly all of those affected report significantly reduced physical performance, which is accompanied by other somatic and cognitive symptoms, in particular concentration problems, shortness of breath, persistent exhaustion, memory problems, headaches, sleep disorders and myalgia. This variety of symptoms corresponds to the manifestations of post-COVID condition described in current research [33,34,35]. The individual burden of specific symptoms was not systematically assessed during anamnesis, limiting conclusions about symptom severity. However, all patients were either still on sick leave or reported ongoing functional impairment. Based on their symptom complexity, they underwent extensive neurological, neuro-psychological, psychological, physio-, ergotherapeutical, pneumological and cardiological examinations. Their referral to the PCC was initiated by the statutory accident insurance to evaluate the need for further rehabilitation or a structured return-to-work process. In a study by Barnekow et al., symptoms categorized as severe were fatigue/exhaustion, loss of sense of smell and taste, concentration and memory difficulties, shortness of breath and pain in the limbs [35].

The importance of acute symptom burden as a predictor of persistent symptoms is consistent with recent international cohort studies and meta-analyses, which describe a dose-like relationship between the number of acute symptoms and the risk of post-COVID symptoms. Cognitive fatigue, dyspnea and limited physical capacity in particular are repeatedly associated with high initial disease activity [36,37]. In line with our findings, a meta-analysis of hospitalized patients also demonstrates that fatigue and dyspnea can persist for more than 12 months, consistent with existing evidence linking these symptoms to severe pulmonary involvement. The authors emphasize that a high initial symptom burden leads to long-lasting functional impairment [38]. However, irrespective of hospitalization status, it must be recognized that the time elapsed since the acute phase may result in recall bias in the reporting of symptoms. Given that COVID was perceived as an existential threat during the early stages of the pandemic, this may have influenced the retrospective appraisal of symptom severity and, consequently, the perceived disease burden.

Although men have been shown to experience more severe courses of acute COVID-19, including significantly higher hospitalization rates, women are more frequently affected by long-term sequelae [39]. In our cohort, the proportion of female participants was elevated, which may partly reflect occupational exposure patterns in healthcare and welfare settings. A large proportion of participants were employed in healthcare and welfare professions, particularly nursing and allied health occupations, which are predominantly female in Germany and experienced a disproportionate burden of occupational SARS-CoV-2 exposure during the pandemic. However, this sex-specific vulnerability is not limited to healthcare workers and has also been documented in the general population [40].

A large proportion of patients in this cohort contracted the initial infection during the first two waves of the SARS-CoV-2 pandemic in 2020. These infections occurred before the availability of vaccines against SARS-CoV-2 in Germany for frontline staff, during a period dominated by the original Wuhan strain [41]. The early variants have been linked to significantly higher rates of severe illness, hospitalization and long-term complications, particularly among frontline healthcare workers [42]. Consistent with our sample, prior research indicates that occupational groups in healthcare, education, social work, trade or transportation experience a higher risk of developing PCS [43,44]. Furthermore, Al-Oraibi et al. identified the nursing profession as an independent predictor of PCS (aOR 1.76; 95% CI 1.26–2.46) compared to medical professions [45]. Overall, the risk of PCS appears to be analogous to the epidemiological pattern of SARS-CoV-2 exposure. Health literacy and greater symptom awareness among healthcare workers may explain the higher PCS reporting among healthcare workers. However, healthcare workers were disproportionately exposed during the early pandemic waves, and since PCS was most frequent and severe following wild-type SARS-CoV-2 infection, this suggests that healthcare workers likely face an elevated risk of developing PCS [21,46,47].

The associations between specific pre-existing conditions and individual symptoms highlight the importance of underlying diseases. In this study, neurological, respiratory, psychiatric, cardiovascular and metabolic conditions appear to modify the course of certain symptom dimensions. In particular, pulmonary pre-conditions (such as asthma, COPD, pulmonary embolism, pneumonia or obstructive sleep apnea) were predictive for dyspnea, a common symptom in PCS [48]. Matsuyama et al. found that pre-existing respiratory conditions and mechanical ventilation during the acute phase were linked to dyspnea lasting up to 12 months [49]. Furthermore, Smith et al. reported that elevated BMI and a prior history of asthma were additional independent factors for dyspnea. PCS-related dyspnea was also more closely associated with fatigue than with pulmonary dysfunction or exertional hypoxia. The authors speculate that there might be two dyspnea phenotypes: a fatigue-dominant type with normal lung function and a pulmonary-impairment type with abnormal pulmonary function, where intrinsic lung defects drive dyspnea [50]. We will conduct further symptom cluster analyses and evaluate pulmonary function tests to investigate the relationship between fatigue and dyspnea in our cohort, as these frequently co-occur and may share underlying pathophysiological mechanisms.

In our analysis, cardiovascular preconditions were predictive of neuromuscular symptoms such as myalgia, which is a common neuromuscular manifestation of PCS [51]. A systematic review identified COVID survivors with pre-existing cardiovascular diseases such as hypertension, congestive or chronic heart failure, myocardial infarction, transient ischemic attack or venous thromboembolism as a high-risk group for developing mild to severe PCS. Typical symptoms in patients with pre-existing conditions included fatigue, dyspnea, chest pain, myalgia and cough, accompanied by mental health conditions such as insomnia, panic disorder, cognitive impairment and depressive disorder [52]. Acute COVID-19 has been associated with electrocardiographic abnormalities, including right and left ventricular dysfunction, diastolic impairment and pericardial effusion. These alterations may compromise cardiac function, increase blood pressure and exacerbate pre-existing hypertension. In addition, sleep disturbances and perceived low quality of life may negatively impact cardiac function, trigger arrhythmias, raise blood pressure and contribute to the development or worsening of hypertension [53].

Metabolic and neurological conditions were also predictive of sleep disturbances, which may reflect underlying dysregulation of the central nervous and autonomic systems. In a small cohort (*n* = 39), Reid et al. showed that patients with neurologic manifestations of PCS had lower sleep efficiency, longer sleep latency and later sleep midpoint compared to age-matched healthy controls (*n* = 71) with no history of PCS. Self-reported cognitive symptoms correlated with the severity of fatigue, anxiety and depression [54]. Such disturbances are thought to exacerbate neurocognitive impairment (e.g., decreased performance in attention and processing speed) and perpetuate a cycle of fatigue, both of which are frequently observed in PCS [54,55,56]. According to a recent review, patients with a history of depression, anxiety disorders, or burnout had a significantly higher risk of persistent symptoms, particularly the development of neurocognitive complaints [57]. However, several studies have highlighted a mismatch between subjective cognitive symptom burden and objective functional assessments, suggesting that underlying psychological conditions, such as depression or somatic symptom disorder, may significantly contribute to the perceived deficits, especially in patients with mild acute courses of COVID-19 [19,58,59,60]. Interestingly, a French study found that persistent physical symptoms up to 12 months after the first wave of the pandemic were more prevalent among those who believed they had experienced a SARS-CoV-2 infection than among those who had a laboratory-confirmed infection [25]. This suggests that psychological factors may significantly influence the persistence of symptoms. We therefore aim to investigate in subsequent analyses whether self-reported cognitive complaints align with objective findings from standardized neurocognitive testing within our cohort.

Our findings are consistent with observations from other European healthcare worker cohorts. In a large multicenter Italian cohort within the ORCHESTRA project, a higher BMI was associated with major acute (35.2%) and post-acute symptoms (14.2%), compared to normal-weight individuals (23.5% and 9.3%, respectively), suggesting that a higher BMI may contribute to a more severe clinical course and an increased risk of a high, persistent symptom burden [61]. Furthermore, reinfections among this healthcare workers cohort have been documented during successive pandemic waves, indicating that repeated viral exposure may contribute to persistent symptoms such as fatigue and cognitive difficulties [62].

### Limitations

Several limitations should be taken into account when interpreting these results. The study population is restricted to individuals referred for specialized PCS evaluation within the BG clinic network who had already reported persistent symptoms and attributed them to COVID-19. Due to registry design and the specialized care structure of these clinics, this represents a clinically selected sample with a high symptom burden. Consequently, the observed frequencies of persistent complaints (e.g., limited physical capacity, 95.6%) likely overestimate those in the broader post-COVID population. Therefore, these findings should not be interpreted as population-based estimates and their generalizability to all individuals with PCS in the working population is limited. However, the observed associations between clinical characteristics and persistent symptoms remain informative for understanding symptom patterns and risk stratification within symptomatic post-COVID populations referred for clinical evaluation.

The recording of symptoms was based on subjective information obtained during clinical consultations within routine health assessments, meaning that reporting bias cannot be ruled out. Additionally, at the time the registry was established, there was no systematic implementation of a standardized, validated functional status instrument, as data collection was embedded in routine clinical health assessments before the widespread use of PCS-specific evaluation tools. Missing data and center-specific variability pose further risks of biased estimates and underestimated standard errors. The heterogeneity of documentation practices, symptom definitions and follow-up procedures in individual BG clinics carries the risk of information bias and varying misclassification. Vaccination status was not available for 42% of the sample due to inconsistent documentation, predominantly among those assessed in earlier registry periods prior to widespread immunization. This reflects temporal patterns rather than selection at the individual level. Accordingly, vaccination status was excluded from the multivariate models as its inclusion would have substantially reduced the analytic sample and introduced temporal confounding. A sensitivity analysis restricted to participants with complete vaccination records was performed, and adjustment for vaccination status did not change the estimated effects of the independent variables meaningfully. Despite statistical adjustment, confounding by indication and other unmeasured covariates cannot be fully excluded. Furthermore, although the registry was established as a longitudinal study, the present analyses are cross-sectional in nature and therefore do not allow conclusions regarding temporal relationships or causal inference. The observed associations between clinical characteristics and persistent symptoms should be interpreted as concurrent relationships rather than causal effects. Future analyses of the registry will address longitudinal effects. It is also possible that changes in diagnostic criteria or patient pathways over time influenced the observed associations. Given the limited number of centers (*n* = 6), random effects variance estimates should be interpreted cautiously, as their precision may be limited. Nevertheless, including a random intercept allowed adjustment for clustering of individuals within centers.

## 5. Conclusions

The present findings indicate that persistent symptoms following work-related SARS-CoV-2 infection are multidimensional and closely linked to acute disease burden, a key predictor of ongoing physical and cognitive impairment. The resulting limitations in occupational and social participation are well documented in the literature. Physical and psychiatric comorbidities appear to contribute to an unfavorable disease course and may promote symptom chronicity. Accordingly, pre-existing conditions should be systematically considered in therapeutic and rehabilitative planning to enhance treatment effectiveness. Within statutory accident insurance, these results underscore the need for differentiated, progression-oriented post-COVID assessments and targeted management of comorbidities to support sustained functional recovery.

## Figures and Tables

**Table 1:** Patient characteristics stratified by sex (N = 1511).

Variables		Male (*n* = 352)	Female (*n* = 1159)	Total *n* (%)
Median Age (IQR)	(Years)	55 (47–60)	53 (44–59)	54 (44–59)
Median Time (IQR) (infection–examination)	(Months)	16 (11–21.5)	16 (12–22)	16 (11–22)
BMI (missing: 42)	<25 (Under/normal weight)	70 (20.6)	419 (37.1)	489 (33.3)
	25–30 (Overweight)	139 (40.6)	332 (29.4)	471 (32.1)
	≥30 (Obesity)	131 (38.5)	378 (33.5)	509 (34.6)
Profession (missing: 15)	Nursing	83 (24.1)	561 (48.7)	644 (43.0)
	Medical	27 (7.9)	53 (4.6)	80 (5.4)
	Therapeutic (e.g., physiotherapy, massage)	10 (2.9)	47 (4.1)	57 (3.8)
	Medical assistance and allied health professions	4 (1.2)	40 (3.5)	44 (2.9)
	Emergency and rescue professions	23 (6.7)	6 (0.5)	29 (1.9)
	Educational/social work	35 (10.2)	285 (24.7)	320 (21.4)
	Administration/other occupations	162 (47.1)	160 (13.9)	322 (21.5)
Workplace (missing: 51)	Hospital	89 (27.4)	416 (36.7)	505 (34.6)
	Medical practice	12 (3.7)	44 (3.9)	56 (3.8)
	Inpatient/outpatient elderly care	35 (10.7)	230 (20.2)	265 (18.2)
	Educational/social welfare	35 (10.8)	299 (26.3)	334 (22.9)
	Other institutions	154 (47.4)	146 (12.9)	300 (20.5)
Pre-existing diseases (missing: 0)	Cardiovascular	166 (47.2)	432 (37.3)	598 (39.6)
	Respiratory	89 (25.3)	284 (24.5)	373 (24.7)
	Neurological	52 (14.8)	242 (20.9)	294 (19.5)
	Psychiatric	56 (15.9)	271 (23.4)	327 (21.6)
	Metabolic/hormonal	98 (27.8)	376 (32.4)	474 (31.4)
	Allergies	104 (29.5)	478 (41.2)	582 (38.5)
	Autoimmune	10 (2.8)	69 (6.0)	79 (5.2)
	Cancer	22 (6.3)	53 (4.6)	75 (5.0)
	Musculoskeletal	75 (21.3)	213 (18.4)	288 (19.1)
	Other ^1^	16 (4.5)	31 (2.7)	47 (3.1)
Number of pre-existing diseases (missing: 27)	0–1	136 (39.7)	415 (36.4)	551 (37.1)
	2–3	131 (38.2)	455 (39.9)	586 (39.5)
	4+	76 (22.2)	271 (23.8)	347 (23.4)
Incapacity to work (missing: 61)	(At time of examination)	166 (49.7)	585 (52.4)	751 (51.8)
COVID vaccination (missing: 638)	Vaccinated	148 (84.1)	612 (87.8)	760 (87.1)
	Not vaccinated	28 (15.9)	85 (12.2)	113 (12.9)
Variant wave initial infection ^2^ (missing: 0)	Wild-type (1 January 2020–28 February 2021)	205 (58.2)	748 (64.5)	953 (63.1)
	Alpha (1 March 2021–31 July 2021)	73 (20.7)	120 (10.4)	193 (12.8)
	Delta (1 August 2021–26 December 2021)	21 (6.0)	73 (6.3)	94 (6.2)
	Omicron (from 27 December 2021)	53 (15.1)	218 (18.8)	271 (17.9)
Reinfections (missing: 0)	At least one reinfection	47 (13.4)	155 (13.4)	202 (13.4)
Hospitalization (missing: 6)	Inpatient (incl. ICU)	111 (31.6)	185 (16.0)	296 (19.7)
	ICU	42 (12.0)	35 (3.0)	77 (5.1)
Number of symptoms at first infection ^3^ (missing: 18)	0–4	73 (21.0)	229 (20.0)	302 (20.2)
	5–9	220 (63.2)	693 (60.5)	913 (61.2)
	10+	55 (15.8)	223 (19.5)	278 (18.6)

Abbreviations: BMI = Body Mass Index; ICU = Intensive Care Unit; IQR = Interquartile Range; ^1^ Other diseases: gastro-, ophthalmo-, hemato-, dermatologic, urogenital or ear, nose and throat (ENT). ^2^ Based on the date of initial infection. ^3^ *n* = 18 individuals were asymptomatic.

**Table 2:** Factors associated with predominant post-COVID symptoms reported during clinical examination.

Variables	Limited Physical Capacity	Impaired Concentration	Dyspnea	Exhaustion/Tiredness	Memory Difficulties	Cognitive Fatigue	Sleep Disturbance	Myalgia
	*n* = 1493	*n* = 1468	*n* = 1447	*n* = 1493	*n* = 1452	*n* = 1493	*n* = 1493	*n* = 1493
	OR (95% CrI)	OR (95% CI)	OR (95% CI)	OR (95% CI)	OR (95% CI)	OR (95% CI)	OR (95% CI)	OR (95% CI)
Sex (Ref: male)	1.67 (0.97, 2.80)	0.96 (0.71, 1.31)	0.92 (0.68, 1.23)	1.01 (0.77, 1.33)	1.29 (0.98, 1.68)	1.16 (0.90, 1.50)	1.24 (0.96, 1.61)	**1.47 (1.14, 1.89)**
Age (Ref: <40)	1	1	1	1	1	1	1	1
40–49 yr	0.92 (0.44, 1.94)	1.12 (0.72, 1.74)	1.0 (0.67, 1.51)	1.28 (0.86, 1.88)	1.09 (0.74, 1.60)	1.18 (0.82, 1.70)	0.77 (0.54, 1.10)	1.11 (0.78, 1.59)
50–59 yr	1.35 (0.67, 2.61)	1.01 (0.69, 1.49)	0.86 (0.60, 1.23)	1.17 (0.83, 1.64)	1.16 (0.83, 1.63)	1.17 (0.85, 1.61)	**1.45 (1.05, 1.99)**	1.08 (0.78, 1.48)
≥60 yr	2.06 (0.88, 4.75)	0.8 (0.52, 1.22)	0.96 (0.64, 1.43)	1.19 (0.82, 1.72)	1.17 (0.81, 1.70)	1.32 (0.93, 1.88)	0.95 (0.67, 1.35)	1.0 (0.70, 1.42)
BMI (Ref: <25)	-	-	1	-	1	-	-	-
25–29.9			1.18 (0.89, 1.58)		**1.44 (1.08, 1.91)**			
≥30			**1.46 (1.08, 1.97)**		**1.42 (1.07, 1.87)**			
Variant (Ref: Omicron)	-	1	-	-	-	-	-	-
Alpha/Delta		1.36 (0.90, 2.06)						
Wild-type		**1.65 (1.17, 2.33)**						
Acute symptoms (Ref: <5)	1	1	1	1	1	1	1	1
5–9	1.18 (0.65, 2.05)	**1.54 (1.12, 2.10)**	**1.69 (1.26, 2.25)**	**1.46 (1.10, 1.93)**	**1.38 (1.04, 1.83)**	**1.47 (1.12, 1.93)**	1.13 (0.85, 1.49)	1.24 (0.94, 1.63)
>10	**4.66 (1.64, 15.3)**	**2.49 (1.54, 4.02)**	**3.1 (2.00, 4.82)**	**3.42 (2.19, 5.34)**	**2.71 (1.79, 4.11)**	**3.52 (2.35, 5.27)**	**2.1 (1.44, 3.06)**	**1.75 (1.21, 2.54)**
Hospitalization (Ref: no)	-	-	**1.62 (1.17, 2.25)**	-	-	-	-	-
Reinfections (Ref: no)	-	-	-	**1.54 (1.07–2.22)**		**1.69 (1.2, 2.39)**	-	-
Neurological condition (Ref: no)	2.08 (1.00, 4.65)	-	-	-	1.34 (0.99, 1.80)	-	**1.32 (1.00, 1.74)**	
Pulmonary condition (Ref: no)	-	-	**1.7 (1.26, 2.29)**	-	-	-	-	-
Psychiatric condition (Ref: no)	-	**1.5 (1.02, 2.19)**	-	**1.58 (1.17, 2.13)**	-	-	-	-
Cardiovascular condition (Ref: no)	-	-	-	-	-	-	-	**1.35 (1.08, 1.70)**
Metabolic condition (Ref: no)	-	-	-	-	-	-	**1.48 (1.17, 1.88)**	-
Number of pre-conditions (Ref: 0–1)	-	1	-	-	-	-	-	-
2–3		**1.61 (1.20–2.16)**						
≥4		**2.35 (1.57, 3.53)**						
ICC	0.08	0.04	0.04	0.06	0.03	0.04	0.06	0.06
Center.SD (Intercept)	0.52	0.37	0.39	0.47	0.32	0.36	0.47	0.46

The table reports odds ratios (ORs) with 95% confidence intervals (95% CIs) for all variables retained in the final multivariable model. Results for Bayesian analysis are reported with 95% credible intervals (CrI), representing the probability that the true parameter lies within the interval. Values in bold indicate statistically significant results (*p* < 0.05). The number of observations differed between the regression models due to the outcome-specific complete case datasets and varying patterns among the independent covariates.

## Data Availability

The dataset presented in this article is not readily available because the data are part of an ongoing registry study. Requests to access the datasets should be directed to the University Medical Clinic Hamburg Eppendorf, Center for Epidemiology and Health Services Research for Healthcare Professionals (CVcare), Hamburg, Germany.

## Supplementary Materials

The following supporting information can be downloaded at https://www.mdpi.com/article/10.3390/diseases14040141/s1: Table S1: Sensitivity analysis including vaccination status for outcome limited physical capacity obtained from Bayesian logistic regression models (N = 863); Table S2: Sensitivity analysis including vaccination status for outcome impaired concentration obtained from generalized linear mixed-effects models (N = 847); Table S3: Sensitivity analysis including vaccination status for outcome dyspnea obtained from generalized linear mixed-effects models (N = 844); Table S4: Sensitivity analysis including vaccination status for outcome exhaustion/tiredness obtained from generalized linear mixed-effects models (N = 863); Table S5: Sensitivity analysis including vaccination status for outcome memory difficulties obtained from generalized linear mixed-effects models (N = 847); Table S6: Sensitivity analysis including vaccination status for outcome cognitive fatigue obtained from generalized linear mixed-effects models (N = 863); Table S7: Sensitivity analysis including vaccination status for outcome sleep disturbance obtained from generalized linear mixed-effects models (N = 863); Table S8: Sensitivity analysis including vaccination status for outcome myalgia obtained from generalized linear mixed-effects models (N = 863); Table S9: Proportion of missing data for variables (N = 1511); Table S10: Bivariate screening analyses for selection of covariates in outcome-specific multivariable models. Bold values indicate *p* < 0.10. Variables meeting this criterion were entered into the initial models.

## Abbreviations

The following abbreviations are used in this manuscript:

AIC	Akaike Information Criteria
ARDS	Severe Acute Respiratory Distress Syndrome
BG clinic	Berufsgenossenschaftliches Klinikum (clinic for occupational accident insurance)
BIC	Bayesian information criteria
CI	Confidence Interval
COVID	Coronavirus Disease
CrI	Credible Intervals
DGUV	Deutsche Gesetzliche Unfallversicherung (German Statutory Accident Insurance)
eCRF	Electronic Case Report Form
ENT	Ear, Nose and Throat
ICF	Informed Consent Form
ICU	Intensive Care Unit
IQR	Interquartile Range
OR	Odds Ratio
PCC	Post-COVID Check
PCS	Post-COVID Syndrome
REDCap	Research Electronic Data Capture
RR	Relative Risk
SARS-CoV-2	Severe Acute Respiratory Syndrome Coronavirus 2
SD	Standard Deviation
STROBE	Strengthening the Reporting of Observational studies in Epidemiology
