# Supplementary material for: Persistent Symptoms After SARS-CoV-2 Infection in a Referred Occupational Clinical Registry: Symptom Patterns and Associated Factors
Source: Diseases. 2026 Apr 9;14(4):141. doi: 10.3390/diseases14040141 (PMC13114690; doi:10.3390/diseases14040141)
Supplement: Supplementary file 1 [file diseases-14-00141-s001.zip › diseases-4165385-supplementary (1).pdf]

Supplementary Material

Title: Persistent Symptoms after SARS-CoV-2 Infection in a Referred Occupational Clinical Registry: Symptom Patterns and Associated Factors

Part I: Sensitivity Analysis Vaccination Status

Sensitivity analysis was restricted to participants with available vaccination data. Within this restricted sample, models with and without adjustment for vaccination status were compared to ensure that any differences in estimates were attributable to vaccination adjustment rather than changes in sample size.

Odds ratios (OR) and 95% credible intervals (CrI) were obtained from Bayesian logistic regression models with a random intercept for study center for the outcome *Limited Physical Capacity*. For all remaining outcome variables OR and 95% confidence intervals (CI) were estimated using generalized linear mixed-effects models with a random intercept for study center.

Supplementary Table S1. Sensitivity analysis including vaccination status for Outcome ***Limited Physical Capacity*** obtained from Bayesian logistic regression models (N=863)

**Variable**	**Vaccination subset OR (95% CrI)**	**Vaccination-adjusted model OR (95% CrI)**
**Sex** (Ref: male)	0.94 (0.41–2.01)	0.94 (0.41–2.02)
**Age 40–49** (Ref: <40)	1.40 (0.55–3.62)	1.40 (0.55–3.57)
**Age 50–59** (Ref: <40)	1.66 (0.72–3.66)	1.66 (0.72–3.68)
**Age ≥60** (Ref: <40)	2.76 (1.01–8.11)	2.76 (1.01–7.90)
**Acute symptoms 5–9** (Ref: <5)	1.45 (0.68–3.01)	1.42 (0.66–2.96)
**Acute symptoms ≥10** (Ref: <5)	5.40 (1.57–21.52)	5.34 (1.55–21.0)
**Neurological condition**	1.76 (0.73–4.88)	1.74 (0.72–4.72)
**Vaccination (yes/no)**	—	1.25 (0.51–2.81)
**Center.SD (Intercept)**	0.39	0.38

Supplementary Table S2. Sensitivity analysis including vaccination status for Outcome **Impaired Concentration** obtained from generalized linear mixed-effects models (N=857)

Variable	Vaccination subset OR (95% CI)	Vaccination-adjusted model OR (95% CI)
**Sex** (Ref: male)	0.99 (0.64–1.54)	0.97 (0.62–1.51)
**Age 40–49** (Ref: <40)	1.33 (0.73–2.43)	1.33 (0.72–2.43)
**Age 50–59** (Ref: <40)	0.96 (0.58–1.59)	0.93 (0.56–1.55)
**Age ≥60** (Ref: <40)	0.88 (0.50–1.54)	0.87 (0.50–1.54)
**Variant: Alpha/Delta** (Ref: Omicron)	1.38 (0.83–2.29)	1.49 (0.90–2.49)
**Variant: Wildtype** (Ref: Omicron)	1.79 (1.17–2.73)	1.99 (1.29–3.08)
**Acute symptoms 5–9** (Ref: <5)	1.51 (0.98–2.35)	1.45 (0.93–2.27)
**Acute symptoms ≥10** (Ref: <5)	2.54 (1.32–4.87)	2.46 (1.27–4.77)
**Psychiatric condition**	1.57 (0.97–2.53)	1.56 (0.97–2.53)
**No of pre-conditions: 2–3** (Ref: 0-1)	1.36 (0.91–2.03)	1.37 (0.92–2.05)
**No of pre-conditions: ≥4** (Ref: 0-1)	1.82 (1.07–3.10)	1.74 (1.02–2.97)
**Vaccination (yes/no)**	—	2.02 (1.24–3.27)
**Center.SD (Intercept)**	0.202	0.170

Supplementary Table S3. Sensitivity analysis including vaccination status for Outcome **Dyspnea** obtained from generalized linear mixed-effects models (N=844)

Variable	Vaccination subset OR (95% CI)	Vaccination-adjusted model OR (95% CI)
**Sex** (Ref: male)	0.77 (0.51–1.17)	0.76 (0.50–1.15)
**Age 40–49** (Ref: <40)	0.97 (0.57–1.65)	0.97 (0.57–1.66)
**Age 50–59** (Ref: <40)	0.90 (0.57–1.44)	0.89 (0.55–1.42)
**Age ≥60** (Ref: <40)	0.98 (0.58–1.65)	0.97 (0.58–1.64)
**BMI: 25-29.9** (Ref: <25)	1.05 (0.71–1.55)	0.99 (0.67–1.47)
**BMI: ≥30** (Ref: <25)	1.16 (0.79–1.70)	1.15 (0.78–1.69)
**Acute symptoms 5–9** (Ref: <5)	1.89 (1.27–2.79)	1.83 (1.23–2.72)
**Acute symptoms ≥10** (Ref: <5)	4.03 (2.28–7.12)	3.86 (2.17–6.84)
**Pulmonary condition**	1.39 (0.95–2.04)	1.38 (0.94–2.03)
**Inpatient treatment**	1.84 (1.14–2.98)	1.93 (1.19–3.15)
**Vaccination (yes/no)**	—	1.76 (1.11–2.78)
**Center.SD (Intercept)**	0.389	0.403

Supplementary Table S4. Sensitivity analysis including vaccination status for Outcome **Exhaustion/Tiredness** obtained from generalized linear mixed-effects models (N=863)

**Variable**	**Vaccination subset OR (95% CI)**	**Vaccination-adjusted model OR (95% CI)**
**Sex** (Ref: male)	1.14 (0.77–1.68)	1.10 (0.75–1.63)
**Age 40–49** (Ref: <40)	1.18 (0.70–2.00)	1.17 (0.69–2.00)
**Age 50–59** (Ref: <40)	1.09 (0.70–1.72)	1.06 (0.67–1.68)
**Age ≥60** (Ref: <40)	1.10 (0.67–1.81)	1.09 (0.66–1.81)
**Acute symptoms 5–9** (Ref: <5)	1.42 (0.97–2.08)	1.34 (0.91–1.98)
**Acute symptoms ≥10** (Ref: <5)	4.26 (2.31–7.86)	4.03 (2.17–7.49)
**Psychiatric condition**	1.46 (1.00–2.14)	1.41 (0.96–2.07)
**Reinfection (yes/no)**	1.35 (0.88–2.06)	1.38 (0.90–2.12)
**Vaccination (yes/no)**	—	2.36 (1.53–3.63)
**Center.SD (Intercept)**	0.311	0.297

Supplementary Table S5. Sensitivity analysis including vaccination status for Outcome **Memory difficulties** obtained from generalized linear mixed-effects models (N=847)

Variable	Vaccination subset OR (95% CI)	Vaccination-adjusted model OR (95% CI)
**Sex** (Ref: male)	1.37 (0.95–1.98)	1.36 (0.94–1.96)
**Age 40–49** (Ref: <40)	0.90 (0.55–1.49)	0.90 (0.55–1.49)
**Age 50–59** (Ref: <40)	1.04 (0.67–1.62)	1.04 (0.67–1.61)
**Age ≥60** (Ref: <40)	1.15 (0.70–1.87)	1.14 (0.70–1.87)
**BMI: 25-29.9** (Ref: <25)	1.18 (0.82–1.72)	1.16 (0.80–1.68)
**BMI: ≥30** (Ref: <25)	1.38 (0.96–1.98)	1.37 (0.96–1.97)
**Acute symptoms 5–9** (Ref: <5)	1.11 (0.75–1.64)	1.09 (0.74–1.61)
**Acute symptoms ≥10** (Ref: <5)	2.16 (1.27–3.67)	2.10 (1.23–3.58)
**Neurological precondition**	1.22 (0.83–1.79)	1.22 (0.83–1.78)
**Vaccination (yes/no)**	—	1.27 (0.81–1.99)
**Center.SD (Intercept)**	0.156	0.164

Supplementary Table S6. Sensitivity analysis including vaccination status for Outcome **Cognitive Fatigue** obtained from generalized linear mixed-effects models (N=863)

Variable	Vaccination subset OR (95% CI)	Vaccination-adjusted model OR (95% CI)
**Sex** (Ref: male)	1.31 (0.91–1.88)	1.28 (0.89–1.85)
**Age 40–49** (Ref: <40)	0.90 (0.55–1.47)	0.90 (0.55–1.47)
**Age 50–59** (Ref: <40)	1.11 (0.72–1.69)	1.09 (0.71–1.67)
**Age ≥60** (Ref: <40)	1.01 (0.63–1.62)	1.01 (0.63–1.62)
**Acute symptoms 5–9** (Ref: <5)	1.39 (0.95–2.03)	1.34 (0.91–1.97)
**Acute symptoms ≥10** (Ref: <5)	3.29 (1.95–5.55)	3.17 (1.87–5.37)
**Reinfection (yes/no)**	1.62 (1.08–2.42)	1.63 (1.09–2.45)
**Vaccination (yes/no)**	—	1.54 (1.00–2.38)
**Center.SD (Intercept)**	0.455	0.448

Supplementary Table S7. Sensitivity analysis including vaccination status for Outcome **Sleep disturbance** obtained from generalized linear mixed-effects models (N=863)

Variable	Vaccination subset OR (95% CI)	Vaccination-adjusted model OR (95% CI)
**Sex** (Ref: male)	1.40 (0.99–1.98)	1.37 (0.97–1.95)
**Age 40–49** (Ref: <40)	0.75 (0.47–1.20)	0.75 (0.47–1.20)
**Age 50–59** (Ref: <40)	1.37 (0.91–2.06)	1.36 (0.90–2.05)
**Age ≥60** (Ref: <40)	1.07 (0.68–1.69)	1.08 (0.68–1.70)
**Acute symptoms 5–9** (Ref: <5)	1.38 (0.95–2.01)	1.34 (0.92–1.96)
**Acute symptoms ≥10** (Ref: <5)	2.37 (1.45–3.87)	2.29 (1.40–3.76)
**Neurological precondition**	1.66 (1.16–2.39)	1.65 (1.15–2.38)
**Metabolic precondition**	1.43 (1.05–1.96)	1.42 (1.04–1.95)
**Vaccination (yes/no)**	—	1.47 (0.97–2.24)
**Center.SD (Intercept)**	0.322	0.329

Supplementary Table S8. Sensitivity analysis including vaccination status for Outcome **Myalgia** obtained from generalized linear mixed-effects models (N=863)

**Variable**	**Vaccination subset OR (95% CI)**	**Vaccination-adjusted model OR (95% CI)**
**Sex** (Ref: male)	1.29 (0.92–1.82)	1.28 (0.90–1.80)
**Age 40–49** (Ref: <40)	1.16 (0.73–1.83)	1.16 (0.73–1.84)
**Age 50–59** (Ref: <40)	1.03 (0.68–1.54)	1.02 (0.68–1.53)
**Age ≥60** (Ref: <40)	1.20 (0.76–1.89)	1.20 (0.76–1.89)
**Acute symptoms 5–9** (Ref: <5)	1.35 (0.93–1.96)	1.32 (0.91–1.92)
**Acute symptoms ≥10** (Ref: <5)	2.10 (1.31–3.38)	2.05 (1.27–3.30)
**Cardiovascular precondition**	1.31 (0.98–1.76)	1.31 (0.98–1.76)
**Vaccination (yes/no)**	—	1.35 (0.89–2.04)
**Center.SD (Intercept)**	0.274	0.282

Part II: Additional Information Concerning Missings & Modeling Approach

Supplementary Table S9: Proportion of missing data for variables (N = 1511).

Variable	N Missing	% Missing
Age category	0	0.0%
Sex	0	0.0%
BMI category	42	2.8%
Profession	15	0.99%
Workplace	51	3.4%
Pre-existing diseases		
Cardiovascular	0	0.0%
Respiratory	0	0.0%
Neurological	0	0.0%
Psychiatric	0	0.0%
Metabolic/hormonal	0	0.0%
Allergies	0	0.0%
Autoimmune	0	0.0%
Cancer	0	0.0%
Musculoskeletal	0	0.0%
Other diseases	0	0.0%
No of pre-existing diseases	27	1.8%
Incapacity to work	61	4.0%
COVID vaccination	638	42.2%
Variant wave initial infection	0	0.0%
Reinfections	0	0.0%
Hospitalization	6	0.4%
No of symptoms at first infection	18	1.2%
Infection-to-assessment interval	0	0.0%

Supplementary Table S10: Bivariate screening analyses for selection of covariates in outcome-specific multivariable models. Bold values indicate $p < 0.10$. Variables meeting this criterion were entered into the initial models.

Variable	Outcomes							
	Limited physical capacity	Impaired concentration	Dyspnea	Exhaustion/ Tiredness	Memory difficulties	Cognitive Fatigue	Sleep disturbance	Myalgia
Age category	**0.048**	0.600	0.914	0.922	0.742	0.361	0.355	0.768
Sex	**0.031**	0.283	0.513	0.392	**0.012**	0.222	**0.008**	**0.001**
BMI category	0.772	0.744	**0.001**	0.693	**0.046**	0.226	0.323	0.623
Profession	**0.086**	0.105	0.252	0.936	0.326	**0.027**	**0.005**	0.171
Pre-existing diseases								
Cardiovascular	0.902	0.156	**0.002**	0.821	**0.020**	0.260	**0.029**	**0.010**
Pulmonary	0.463	**0.054**	**0.001**	0.441	0.411	0.105	0.905	0.499
Neurological	**0.062**	**0.022**	0.334	0.296	**0.014**	0.100	**0.016**	0.313
Psychiatric	0.111	**0.001**	0.835	**0.001**	**0.054**	**0.057**	**0.023**	0.707
Metabolic	0.791	**0.005**	0.185	0.258	0.180	0.534	**0.001**	0.249
No of pre-existing diseases	0.418	**0.001**	**0.001**	0.148	**0.001**	**0.026**	**0.003**	**0.049**
Variant wave initial infection	0.401	**0.092**	0.510	0.323	0.229	0.692	0.157	0.285
Reinfections	0.154	0.364	0.694	**0.025**	**0.083**	**0.004**	**0.052**	0.409
Hospitalization	0.326	0.498	**0.001**	0.204	0.565	0.448	0.545	0.954
No of symptoms at first infection	**0.003**	**0.001**	**0.001**	**0.001**	**0.001**	**0.001**	**0.001**	**0.001**
Infection-to-assessment interval	0.301	**0.028**	**0.064**	**0.049**	**0.076**	**0.001**	**0.026**	0.469
Prior use of rehabilitation measures	0.153	**0.003**	0.782	0.399	**0.017**	**0.001**	**0.008**	**0.003**
